# State of Research Quality and Knowledge Transfer and Translation and Capacity Strengthening Strategies for Sound Health Policy Decision-Making in Palestine

**DOI:** 10.3389/ijph.2021.620425

**Published:** 2021-08-02

**Authors:** Mohammed AlKhaldi, Hamza Meghari, Irene Anne Jillson, Abdulsalam Alkaiyat, Marcel Tanner

**Affiliations:** ^1^ University of Basel, Basel, Switzerland; ^2^ Swiss Tropical and Public Health Institute, Basel, Switzerland; ^3^ Faculty of Medicine, McGill University, Montreal, QC, Canada; ^4^ Council on Health Research for Development, Geneve, Switzerland; ^5^ Faculty of Medicine and Health Sciences, An-Najah National University, Nablus, Palestine; ^6^ University College London (UCL), London, United Kingdom; ^7^ School of Medicine, Georgetown University, Washington, DC, United States

**Keywords:** Heath research system capacity, research quality, knowledge transfer and translation, health decision-making, Palestine

## Abstract

**Objectives:** Over the last 2 decades, the World Health Organization (WHO) has proposed a global strategy and initiatives to establish a Health Research System (HRS) focusing on Health Research Quality and Standardization (HRQS), Health Research Knowledge Transfer and Dissemination (HRKTD), and Health Research Translation and Utilization into Health Care Decisions and Policies (HRTUDP). Despite the increase in health research productivity over the past several decades, HRS Capacity (HRSC) in Palestine and in the Middle East and North Africa (MENA) region has rarely been objectively evaluated. This study aims at eliciting the perceptions of HRS performers in Palestine in order to understand the status of HRSC, identify gaps, and generate policies and solutions capable of strengthening HRSC in Palestine.

**Methods:** Key informants from three sectors, namely government, academia, and local and international organizations, were selected purposively based on different sampling methods: criterion, critical case, snowball, and homogeneous sampling. Fifty-two in-depth interviews with key informants and a total of fifty-two individuals, participating in six focus groups, were conducted by the principal investigator in Palestine. Data were analyzed by using MAXQDA 12.

**Results:** The overall pattern of the Palestinian HRSC is relatively weak. The key findings revealed that while HR productivity in Palestine is improving, HRQS is at an average level and quality guidelines are not followed due to paucity of understanding, policies, and resources. HRKTD is a central challenge with both a dearth of conceptualization of translational science and inadequate implementation. The factors related to inadequate HRKTD include lack of awareness on the part of the researchers, inadequate regulatory frameworks and mechanisms for both communication and collaboration between and among researchers and policy-makers and clinicians, and lack of availability of, and credibility in, systematized and reliable HR data. Despite the limited knowledge translation, in general, HRTUDP is not considered an essential decision-making methodology mainly due to the lack of interface between knowledge producers (researchers) and users (policymakers), understanding level, HR credibility and availability of applied research, and governance, resources, and political fluctuations. Recommendations to strengthen HRS in Palestine include: a consolidated research regulatory framework and an effective capacity strengthening strategy overseen by Palestinian authorities; the promotion of HRQS and concepts and practices of translational science; and, most importantly, the use of findings for evidence-based policies and practice.

**Conclusion:** Strengthening HRSC is both an imperative step and an opportunity to improve the Palestinian health system and ensure it is based on research evidence and knowledge. Building a successful HRS characterized by capacities of high-quality research and well-disseminated and translated knowledge is a prerequisite to effective health systems and services. This can be achieved by political commitment to support such strengthening, a consolidated leadership and governance structure, and a strong operational capacity strengthening strategy.

## Introduction

Health research has been recognized as an essential tool in addressing health and development challenges, yet the current capacity of many developing countries to conduct relevant, high-quality, and rigorous health research and disseminate and translate findings into practice remains limited [1, 2]. The knowledge produced by health reseacch is a global public good, indispensable for improving the health of individuals and populations, developing evidence-informed policies, and enhancing the performance of health systems [3, 4]. The establishment of robust Health Research Systems (HRS) requires that nations develop and sustain the capacities necessary to produce research, disseminate findings, apply them in policy and practice, and to evaluate the impact of research on health outcomes [5]. According to the framework of Pang et al., HRS aims to advance scientific knowledge and ensure the use of knowledge to improve health and equity. The significance of Pang’s framework is that it adopts a systems perspective towards HRSs and serves as a foundation for constructing a practical approach to describe and analyse HRSs. There is broad consensus among the scientific community that high-quality knowledge should be generated and appropriately transferred and translated to support health decision-making and policy formulation, contributing to effective and efficient decisions by planners, policy-makers, and professionals [2, 4, 6].

Consequently, health research quality and standardization (HRQS), health research knowledge transfer and dissemination (HRKTD), and health research translation and utilization into decisions and policies (HRTUDP) are the key operational and functional capacities of a HRS. These three capacities featured in conceptual HRS frameworks compose a solid base for building and advancing any HRS [7–10]. These frameworks serve as a basis for understanding health systems through a wide-range analysis [11–13]. Similar studies have shown the importance of strengthening these capacities from a system-wide approach [14–17]. Evaluating and strengthening the capacities of a HRS are essential to improve health outcomes and address global health challenges not only in developing countries but also worldwide [14, 18].

HRS capacity strengthening (HRSCS) is an ongoing process internationally. Certain efforts to strengthen HRS capacities, supported by the World Health Organization (WHO), the Council on Health Research for Development, and the Global Forum on health research and other agencies, underline this process explicitly [19, 20]. However, HRSCS remains one of the world's unmet challenges in developing countries [20], in particular in the context of the 10/90 gap: less than 10% of global resources for health research address health problems in developing countries, where over 90% of preventable deaths occur [21]. The 10/90 gap has remained wide for nearly three decades and has had significant consequences; not only is HRSC not strengthened in low-income countries, but there are, as a result, relatively relevant findings of major gaps in HRSC that can also be applied to policy processes, clinical practices, and monitoring and evaluation of research results and outputs generally and in sub-Saharan Africa [22].

HRSCS strategy has been implemented worldwide to address these gaps to improve the ability of developing countries to overcome the persistent and disproportionate burden of disease that they face. Notably, although the terms “capacity-building” and “capacity-strengthening” are often used interchangeably, there is a distinction: the first refers to the establishment of a research infrastructure, while the second denotes the enhancement of pre-existing infrastructure [23]. The HRSCS strategy has gained substantial attention where donors have invested in a capacity-strengthening approach and are therefore increasingly interested in evaluating the benefits of their investments in health research [7]. Globally, different initiatives and efforts have highlighted the importance of HRSCS through collaboration and harmonization [14]. The ESSENCE initiative is an example of collaboration, allowing donors/funders of HRCS to identify synergies, establish coherence, and increase the value of resources and activities for health research. This paper suggests that embracing the framework of HRSCS is useful along with the adopted frameworks of a HRS.

Across the Middle East and North Africa region, health research faces critical deficits in governance, resources, and capacity in terms of knowledge production, dissemination, and application [11, 22]. Although similar deficits exist in Palestine (the West Bank, Gaza Strip, and East Jerusalem) the context is different. The first Palestinian National Authority was established in 1994 and the responsibility of health was handed over from the Israeli military occupation to the Palestinian Ministry of Health. Since then, Palestinians have remained under occupation that continues to adversely impact all life aspects and public service systems and institutions, in particular the health system and research institutions/universities. The health system in Palestine is fragmented and lacking in resources, infrastructure, evidence-based-policies, and governance as a result of the protracted Israeli occupation and intra-Palestinian divide. Four healthcare providers constitute the health system in Palestine: the Ministry of Health as regulator and main provider, the United Nations Relief and Works Agency for Palestine Refugees in the Near East (UNRWA), private sector organizations, and Non-Governmental Organisations (NGOs) [50].

Insufficient health knowledge, for example, sheds light on the reality of health research capacities in Palestine. Although research production is increasing [24], the extent of its capacity is still an unexplored challenge and constitutes an issue of concern [15–17, 24–26]. Understanding HRSC is a central step towards strengthening the following key elements: the abilities of individuals, institutions, and country to perform health research functions; defining national priorities; solving national health problems; and utilizing the results of research in policy-making and programme delivery [22]. Furthermore, because Palestine is an external aid-dependent state, it faces the challenge of the inadequate role of local and international stakeholders to support both health research capacity strengthening of individuals and institutions, and support of research itself. The role of donors needs to be promoted and harmonized and any resources and aids should be effectively used [27–29].

This study of HRSC in Palestine, which is consistent with WHO’s strategic directions on HRS development, could also inform plans for the strengthening of HRS capacities at the regional level. It aims at understanding and exploring the status of HRS capacities in Palestine in order to identify gaps and solutions for strengthening HRS. This is the first study to explore HRSCS in Palestine and to outline a clear and comprehensive plan for building a sustainable and rigorous national HRS that is well capacitated in Palestine. A vital assumption of this study is that building or strengthening HRSC in Palestine is an investment decision and an opportunity that will advance not only health but also other development sectors.

Specific objectives:

1. Assess the actual status, gaps, and opportunities of HRS capacities in Palestine.
2. Examine HRS potential to recognize the three vital capacities and competencies: HRQS, HRKTD, and, ultimately, HRTUDP.
3. Generate more insights and solutions to improve HRSCS and address its gaps.

## Methods

This study is part of a more comprehensive systems analysis that used a mixture of qualitative tools and strategies to ensure knowledge saturation level, active participation, and adequate representation. This mixed purposive sampling was as follows: first, criterion sampling made it possible to select participants who could provide specific information on certain study topics. Second, critical case sampling targeted experts to give critical and factual information on the topics under investigation. Third, snowball sampling determined other suitable participants, as we were aware that there were likely other key informants that were not known to us at the outset of the study. Finally, homogenous sampling brought together participants from a similar background and with similar experience. Inclusion and exclusion criteria were established to guide the selection process clearly. The descriptive qualitative research design, methods, and instruments used were typically similar to previous local studies that have dealt with other components of HRS and have been carried out in Palestine [16]. Participants were purposively selected based on advance knowledge and experts’ consultations. Participants were selected from three sectors in the health field: governmental health institutions, schools of public health, and major local and international health agencies. A total of 52 in-depth interviews (IDIs) and six focus group discussions (FGDs) were conducted in Palestine either face-to-face or remotely. A topic interview guide was used. To explore the participants’ answers of the research question, the guide was designed to cover the main areas of the HRS, including three health research capacities: HRQS, HRKTD, and, HRTUDP. The principal investigator, a middle-aged, male Palestinian, phoned and emailed potential participants and provided them with a copy of the study information sheet. Participants who did not respond to the initial contact received another call and email a couple of weeks thereafter. Data collection was performed by a research-trained team, qualified local postgraduates graduates in public health, and supervised and coordinated by the principal investigator. Data were audio-recorded in the native language Arabic, translated into English and transcribed into Microsoft word sheets at the same time, and precisely revised, checked, and cleaned for accuracy. Thematic and content approaches were applied using MAXQDA 12 (VERBI GmbH, Berlin), a software package for qualitative data management and analysis. All these procedures, along with data revision and coding for IDIs and FGDs, were performed by the principal investigator.

The approach used in collecting, managing, and analyzing data was also typically adopted in previous similar qualitative studies in Palestine. This study, as well as those studies, followed the Gold Standard COREQ (Consolidated Criteria for Reporting Qualitative Research) criteria. These criteria were applied in the stage of designing instruments, sampling, implementing, and reporting the study findings. The main domains of COREQ in both instruments were considered where elements of) research team and reflexivity, study design, and analysis and findings were particularly reported in this paper and other relevant published studies alike [15–17].

## Results

The study participants had diverse backgrounds and leadership positions. The majority were educated to doctoral level and held more than 20 years of experience, particularly those participants from NGOs. One third of the participants were female. Participants and their institutions were distributed as follows: 18 experts from eight academic institutions, 19 participants from 15 NGOs (10 local and five international), and 15 participants from governmental institutions. The overall responses were obtained from 104 experts who are involved in the HRS. Apart from the socio-demographic characteristics of the participants that were previously presented in other relevant studies, the responses covered findings in three key areas of HRSCS:

1) Health research quality and standardization (HRQS).
2) Health research knowledge transfer and knowledge translation (HRKTD).
3) Health research translation and utilization into decisions and policies (HRTUDP).

### Quality and Standardization in Health Research

Table 1 compares the pattern of health research in Palestine against the best quality practices and standards of international research. Findings are rather diverse and were categorized into two groups. The perceptions of this section were divided and categorized into two groups. The first group of experts perceived that research standardization, guidelines, and quality in Palestine are satisfactory and considerably improved. This is clearly outlined by one government participant: “*yes, at the publishing stage where international journals have rigorous guidelines*”. The second group of experts viewed HRQS as on a less than satisfactory level, and considered this issue a big gap and a serious problem. This is clarified by an NGO expert, who said: “*there is an immense number of health research with a lack of quality*”.

**TABLE 1:** Health research quality and standardization (HRQS). Capacity of research quality and knowledge transfer and translation in Palestine: a call for sound decision-making, Palestine, Eastern Mediterranean Region, 2021.

HR quality and standardization (HRQS)			
Theme sector	Theme 1: The status of HRQS	Theme 2: Limiting the HRQS	Theme 2: Enhancing the HRQS
**Gov**	- It is standardized and research actors are qualified	- Unsupportive environment and culture, poor capacity building programmes, weakness of school curriculum, the absence of clear policies and agreed priorities—our abilities, experiences and will are very limited—inadequate funding—lack of experts in research—shortage of data that observe the national productivity of research—HR time, effort and cost—lack of specialized national research centre	- Addressing carefully these gaps—need more budgets, good research management, coordination among all stakeholders—capacities and experiences need to be empowered - encourage academic exchange programmes with others—self-development, sufficient resources, and political support—formulate laws and effective policies will increase our research quality- education programmes in scientific research
	- Most of research meets the international HRQS		
	- We have a quality education system		
	- Research is of high quality, standardized, and meets HRQS		
	- Some research meets the international standardization when it gets published in high impact factor		
	- Research relies on international standardization endorsed by WHO, but we cannot say that it is efficient and standardized		
	- We attempt to meet the HRQS		
	- Barely meets the HRQS criteria, some researchers do high-quality research. We have a lot of research published internationally		
	- At the publishing stage yes where international journals have rigorous guidelines		
	- HRQS on an average level and only for promotions		
	- In fact, I don’t know and it is hard to judge		
	- No, every university has different ways of research		
**Acad**	- Yes, academia has high quality and reaches international guidelines	- Noncompliance with the international research guidelines—brain drain and time constraint—research performed for personal purposes—lack of resources and infrastructure—research plagiarism and researchers bias—weak researchers competencies and skills in different research expertise—gaps in research design, methods, analysis, data quality, and interpretation—lack of international experiences—lack of fund and official sponsorship—lack of good journal accessibility—lack of experimental research—lack of centres of excellence—lack of HRQS monitoring—a gap in the schools’ curriculum	- A good investment in research productivity—allocate appropriate financial support and resources—overcome plagiarism and promote research objectivity—research should be focusing on priorities—systematic capacity building programmes to develop research leaders’, experts’, and postgraduates’ skills in research critical thinking, design, writing and publication—pay attention to the international orientation to exchange knowledge and expertise—effective research policies addressing HRQS
	- Not a lot but some research performed with good quality		
	- We often publish properly		
	- There are some good research with high quality, generally, research has a moderate quality and high quality often done by foreign co-authors		
	- Relatively, we have researchers produce research with good quality		
	- Research quality is variable, some are good and some bad		
	- Majority research has poor quality with a huge gap with the international guidelines		
	- Our research quality is not good enough despite many good publications		
	- Quality is low and not comparable with international standardization		
	- No clear national policy includes guidelines		
	- No, research production is very low quality and quantity, does not meet HRQS		
**NGOs**	- Research is conducted in good quality but still weak against international standard	- Research is unsystematic and lack of strategic research policies or unified body—shortage of resources and facilities—low researchers qualities and skills—inattention and unwillingness to research—quality data but disorganized, unanalyzed and not used in practice—discontinuity of the research process—lack of orientation and linkage with the international research—lack of local high-impact journals—a weakness of institutional research review—duplication in research activities—a gap in the education curriculum of the health professions—research performs for individual not society goals	- More exposure to the international research experiences—investment in the lancet palestinian Health alliance to expand our research capacity and expertise—research teams are essential—research should meet the local needs—An annual national forum for research—Dynamic research monitoring and evaluation system in Palestine—unifying the research concepts, methods, priorities, practices, uses and guidelines.—Promote the use of communication and technology—capacity building programmes in research concepts, methods, and good practices
	- It is satisfying, high quality, and based on scientific methods		
	- Research quality is improving and has a long way to go		
	- Some quality exists but researchers striving to get high quality		
	- It is varying but research quality is a real problem and still perceived weak		
	- Research is not of high quality yet and does not meet international guidelines		
	- Poor quality, it needs more improvement		
	- An immense number of research with a lack of quality		
	- We still have not reached the required research quality		
	- Never, but some individual attempts having reached the quality exist		

The findings indicate that the most frequent gaps facing HRQS can be sorted into two groups: 1) institutional and environmental gaps and 2) gaps related to research and researchers. The first includes the lack of health research policies and priorities, resources and capacity, the lack of specialized excellence research centres, and weak institutional review of research. The second group of gaps comprises deficit in researchers’ culture and attitudes and linkage with the international research landscape, the scarcity of articles published in prestigious high-impact local journals and access difficulties, the weakness of curricula in university health sciences education, and research individualism of research activities.

The respondents collectively identified five recommendations to strengthen HRQS:

1) Strengthening political will to support agreed-upon health research policies and priorities, with a focus on the quality of research.
2) Developing mechanisms to facilitate greater openness to international health research expertise, strengthening partnerships, and exploiting technology and communication facilities for exchange programmes.
3) Systematic capacity building and education programmes to increase stakeholders’ knowledge and competencies about fundamental topics of good research quality such as design, methods, analysis, writing, and publication and dissemination.
4) Providing support for health research teamwork and investing in existing research initiatives such as The Lancet Palestinian Health Alliance, which, as an excellent platform, encourages all research in Palestine to be rigorously designed, implemented, reviewed, and disseminated.
5) Establishing a national monitoring and evaluation mechanism for HRQS.

### Health Research Knowledge Transfer and Dissemination

Table 2 presents the findings for the capacity for health research knowledge transfer and dissemination (HRKTD). The study identified a high-level of agreement among the respondents concerning HRKTD with three themes emerging from the data: the main hindrances to HRKTD, improvements to HRKTD. and prospects to be invested. IDIs and FGDs consistently showed a consensus on the unsatisfactory level of HRKTD and non-systematic and ineffective sharing of evidence, with noticeable descriptions including, for example, the following: “*completely dissatisfied*”, “*not performing well and below the required level*”*,* “*barely transferred or shared*”*,* “*almost paralyzed*”, and “*poor and limited*”. Participants in government FGDs described the HRKTD in negative terms, with health research outputs unused and remaining “on the shelves”. Another small number of participants, mainly academics and government experts, expressed that the HRKTD pattern is often good and growing.

**TABLE 2:** Health research knowledge transfer and dissemination (HRKTD). Capacity of research quality and knowledge transfer and translation in Palestine: a call for sound decision-making, Palestine, Eastern Mediterranean Region, 2021.

HR knowledge transfer and dissemination (HRKTD)			
Theme sector	Theme 1: Limiting factors of HRKTD	Theme 2: Enhancing factors of HRKTD	Theme 3: Opportunities to build on
**Gov**	- Lack of a unified system and policy and HRKTD tools (e.g,. journals and periodicals) with lack of advanced technology infrastructure	- Effective HRKTD and communication mechanisms, solid political will, and rewarding measures—integral-institutionalized system by activating a unified body and building research strategy—resources and incentives are needed—Divide stakeholders’ roles—raise the awareness of HRKTD—real C&C should be enhanced and promote a national scientific-policy dialogue between researchers and decision-makers—a need for an effective and reliable central computerized information system—allocate sufficient resources	- Plenty of published and unpublished research—certain connections and cooperation—well-developed health information unit which has a good collecting method—presence of the lancet palestinian Health alliance and palestinian national institute of public Health—excellent students and experts—some respectable HRKTD academia attempts
	- Weak recognition and immature sharing culture		
	- No specific central HRKTD platform with selective unsystematic HRKTD and unclear roles		
	- Miscommunication and lack of coordination and cooperation		
	- Data inaccessible and unsystematic		
	- Limited HRKTD at micro-institutional while absent at the macro-national level with research redundancy and discontinuity		
	- Disregard to share research outputs with personal desires		
	- Officials’ inattention to use research as a decision-making tool		
	- Research from academia is not disseminated and unexploited		
	- Lack of intellectual integrity and rights		
	- Conducted research is rarely needs-driven		
	- Lot of descriptive research and weak applied research and deficit of specialized research (surgery, internal medicine, etc.)		
**Acad**	- The absence of HRKTD mechanisms	- Coordination, partnership, teaming up, and agreed vision and policies—incentives focus on research quality, credibility, publication and HRKTD abilities—HRKTD platform or communication such knowledge for policy, agreed database, encourages dialogue and research-decision making the linkage—More investment on HRKTD, and a supervisory inclusive body—palestinian national institute of public Health to lead a proper HRKTD—institutional HRKTD means and encourages Arabic publications and specialized conferences —Decision-makers involvement in research and supportive climate and research centres with information accessibility (e.g., libraries, labs, training)—Awareness of role-playing in research and train human resources in HRKTD	- Good HRKTD by many academic institutions—a critical function to get decisions informed by evidence—some universities implemented campaigns on HRKTD—plenty of research—palestinian national institute of public Health could be an HRKTD platform—Human resources with good potentials—WHO and private sector funding role
	- Research either for personal interests or getting funding		
	- HRKTD is only limited to workshops and conferences without a well-informed decision makingPublication incompetence and cost, time limit and academic overloads to perform HRKTD		
	- Uncompleted researchers’ role with lots of unpublished, not disseminated and underutilized research		
	- Resources and fund insufficiency		
	- Communication gaps and researchers are not involved in the decision-making		
	- Unreliable, inaccessible and not well-analyzed data which is descriptive and lack of local good journal		
	- Some HRKTD performed for graduation or personal needs		
	- The difficulty for decision-makers to read and understand English publications and knowledge		
	- Decision-makers are not interested in evidence		
	- Lack of role-play culture among producers and users		
	- Policy-makers’ reliance on internal reports		
**NGOs**	- Lack of a regulating body, leadership, and research credibility and quality	- Ministry of Health should establish a clear governance structure and policy (HRKTD committee) to ensure effective decision-making based on research—More attention to HRKTD and research importance and evaluation—promote team working and coordination and cooperation—set a dynamic and structured HRKTD platform, use technology and encourage policy dialogue—Annual expanded congress to share all relevant national research outputs—the scientific publication needs to be encouraged	- Medical Aid for palestinians—UK- institute of community and public Health partnership in the lancet palestinian Health alliance as the best platform for HRKTD—High experts and international agencies—Advanced technology and distance learning—international publications by local researchers—local conferences
	- Selective HRKTD not inclusive with lack of linkages and coordination among producers and users and institutions		
	- Lack of local high-impact journals (e.g., Arabic), decent conference, and libraries		
	- Inattention to read research findings		
	- Researchers’ incomplete role in HRKTD		
	- Poor roles of ministry of Health to lead and academia to feed		
	- Disorganized, inaccessible, and monopoly of database		
	- Lack of structured and agreed HRKTD platform		
	- International connections are limited		
	- Blockage, political conditions, and individual desires and donors interests impede HRKTD		
	- Lack of publication competence and writing an informative policy analysis		

Six gaps were identified by experts concerning HRKTD:

1. The lack of a regulatory framework, policy, resources, and poor communication and coordination between producers and users, with very weak international networking.
2. Gaps related to the process, such as the immature culture of sharing and lack of tools and mechanisms, particularly with regard to conferences, local journals, periodicals, workshops, libraries, and platforms. Moreover, HRKTD is selective but non-inclusive, meaning that it is limited to micro-institutional and individual levels but not endorsed or practiced in the national policy sphere.
3. Health research is carried out for researchers’ personal purposes and/or donors’ very specific agendas, and is not necessarily addressing the needs of the Palestinian health sector and population.
4. Health research is an incomplete performing process, from the inception of a research idea to the translation of the outputs, and the role and skills of researchers in the dissemination and translation are lacking.
5. Decision-makers’ rely on their internal reporting systems; they are not well evidence-oriented and, thus, the concept of evidence-based decision-making is not practiced.
6. Data inaccessibility, a high degree of disorganization, and a tendency towards the monopolization and control of the data collected by researchers or inability to share the national vital statistics and data with everybody transparently and systematically, as well as blockages and barriers due to political instability.

To address these barriers, respondents made some suggestions to advance HRKTD. The most substantial action plan is obtaining political support to establish a national body to guide health research policy, to mobilize resources, to delineate roles of stakeholders, and to enhance coordination. It is also necessary to increase the awareness and skills of researchers and decision-makers regarding HRKTD. Participants suggested, for example, to:

- Apply technology and interactive facilities;
- Invest in advancing HRKTD mechanisms and tools to enable both science and decision-making communities, to meet for sustained and reciprocal dialogue focusing on the centre for Knowledge to Policy, the Strategic Policy Platform, and the Research-Decision-making Lab^1^;
- Ensure that the information and databases systems are organized, accessible, and transparent; and
- Encourage and support a spirit of research teamwork and incentives to encourage publication.

Interestingly, the experts suggested opportunities that, if utilized, will foster the advancement of the HRKTD process. These included for example: the availability of an abundance of published and unpublished research by Palestinians; existing partnerships, active health research bodies and organizations, excellent experts and students, and relevant academic research flagships or initiatives such as Palestine 2030-Demographic Change: Opportunities for Development.

### Health Research Translation and Utilization Into Decision and Policies

This last section explores the pattern of research translation and utilization into decisions and policies (HRTUDP) in the Palestinian health sector. As Table 3 illustrates, there was a considerable concordance among experts’ perceptions expressed in both IDIs and FGDs that the outputs of health research are not inherently and consistently applied and relied upon in the decision-making process. From their perspective, the spectrum of echoed descriptions ranged as follows: health research in Palestine has “*no application*”*,* is “*weak and poorly-translated*”*,* “*disappointing*”*,* “*un-embedded*”*,* “*ineffective and inappropriate*”*,* “*producer-user huge gap*”*,* “*improper knowledge transfer and dissemination*”*,* “*HRTUDP received executive*’*s inattention and it is not a tool in decision-making*”*,* “*HRTUDP is an unmet issue*”*,* and “*most of the decisions are not evidence-based*”. On the other hand, a limited number of participants believed that there are known practices to effectively translate research findings into policies and practices in Palestine. These practices are carried out on an individual, selective, occasional, and much interest-driven, as well as not well-structured way, in the health decision-making process. However, very few experts expressed their lack of knowledge and awareness about the HRTUDP and its implementation.

**TABLE 3:** Health research translation and utilization into decisions and policies (HRTUDP). Capacity of research quality and knowledge transfer and translation in Palestine: a call for sound decision-making, Palestine, Eastern Mediterranean Region, 2021.

Health research translation and utilization into decision and policies (HRTUDP)			
Theme sector	Theme 1: Limiting factors of HRTUDP	Theme 2: Improving factors of HRTUDP	Theme 3: Opportunities to build on
**Gov**	- Each entity has its own evidence without sharing them with others with poor culture and attitude on research	- Agreed policy, coordination, and agreed priorities for results application—acad. And gov. Communication and developing researchers capacity—reactivate journals clubs to review research findings to be utilized—Decision-makers must be convinced of research evidence in planning and decision-making—Awareness of evidence-based decision-making - systematic policy workshops discuss all implemented research—clear and regular policy briefs to decision-making and planning bodies	- Palestinian national institute of public Health existence and role to take this mandate—the supreme palestinian Health council can play an important role
	- Financial shortage and poor qualification		
	- Gaps in sectorial coordination, communication, and conflicting interests among producers and users		
	- Ineffective evidence and knowledge dissemination, researchers do not share their results		
	- State management changes hinder research translation		
	- Policy-makers preoccupation to read policy briefs with poor quality of research		
	- A plethora of unused information and knowledge		
	- Plenty of descriptive research rather than experiment		
	- The absence of common body implements research outputs		
**Acad**	- Schools’ research outputs are untapped and unused with ineffective dissemination among departments	- An integrated system adopts the research routine translation process and urges decision-makers to be research-oriented and develop their capacities in evidence-based practice—researchers’ and policy-makers’ communication and involvement for effective, efficient and timed translation into decision-making—training capacity building to raise awareness and improve skills on HRTUDP—encourage dissemination through organizational plan supported by a high national scientific research body—new policies dedicated to evidence-based practice and collaborative work to involve all players on how to translate evidence into decision-making—sectorial research-policymaking coordination and cooperation based on agreed priorities in research topics selection, conduction, and dissemination	- Successful attempts which are evidence-based (such as non-communicable diseases screening)—WHO explicit role to develop HRS and seize lancet annual meetings—without evidence-based decisions, big losses, e.g., waste resources, unimproved health, and incorrect decisions will rise - Health system and care will be met and improved—birziet University and the lancet palestinian Health alliance achieved some success
	- HRTUDP is not a methodology of state policy-making with lack of policy-makers’ research orientation and their dependency on political inputs rather than evidence enforced by political and donors’ agendas		
	- No transparency and immaturity of evidence-based practices culture with limited resources		
	- Lack of communication between researchers and decision-makers due to unshared knowledge through clear interpreted findings		
	- Unpublished research, research not priorities-based and not health system needs-oriented		
	- Time limitation to academics for dissemination		
	- NGOs are dependent and subject to the donors’ wills		
	- Lack of experimental studies and research quality and credibility is an issue		
	- Research does not address health improvement and is mainly personal-interest driven		
	- Health actions are spontaneously performed not based on research with contradiction goals in obtaining the funding		
**NGOs**	- Lack of policy-makers’ awareness and interest in research	- A clear structure to guide research, foster knowledge transfer and translation, a solid link between researchers and policy-makers—MoH should embrace evidence-based decisions in policy-making processes—support to encourage human resources—a body to implement evidence translation, local-international networks to benefit from their experience and to get accessibility—politic tensions should be separated from development decisions—partnerships provide empowerment programmes, allocate resources, academia-state integration—research culture should be enhanced and integrated into the decision-making process—All health interventions need to be based on evidence and aligned with research priorities	- Major improvements in post—and ante-natal care are based on evidence—palestinian national institute of public Health to lead improving evidence-based practice and knowledge transfer—Utilizing the presence of scientific research council
	- Lack of clear research system and agreed research priorities and policy		
	- Unmonitored decision-making and lack of policy informing and briefing skills with poor communication and dissemination among stakeholders		
	- Produced evidence does not reflect the national priorities and it not connected to the society’s needs		
	- Lack of research quality and data credibility		
	- The negative impact of social, political and economic instability on decision-making		
	- Research is a personal interest with no influence on decision-making		
	- Most of research done in health schools is neglected and unutilized		
	- The inability of state legislative boards to use research findings in their decision-making, good research selection is an issue		
	- The abundance of evaluative and statistical studies with a deficit of experimental ones		

The study found critical gaps that adversely affect the translation of research findings to policy and practice. These gaps are divided into three categories according to the prominence in the responses: 1) conceptual, 2) technical, and 3) gaps related to the health system and HRS at large. Conceptually, decision-makers and policy-makers are not yet research-oriented, with a lack of knowledge and lack of culture of evidence-based decision-making and knowledge-informed policy. Technical gaps revealed major communication and coordination gaps between researchers and decision-makers. There were poor knowledge transfer and dissemination and data sharing mechanisms and tools, issues concerning health research quality and credibility, the fact that research carried out by individual researchers does not reflect crucial national priorities, and plenty of descriptive research but a paucity of government investment in applied and experimental research. This investment (which currently does not exist in Palestine) should be a central item of GDP spending managed by a consortium of institutions, such as the Ministries of Finance, Higher Education, Health, National Banks and Funds, etc. Health research produced by academics does not receive serious State attention. The last group of shortcomings for health system and HRS was the absence of a unified regulatory framework, absence of HRS policies and priorities, lack of resources dedicated to health research, institutional instability and management changes, and national political and economic conditions such as financing restrictions, blockage, and loss of sovereignty.

For the initiation of a dynamic process for knowledge transfer and translation in decision-making and policy formulation of the health system, respondents recommended different proposals that fall into two categories: structural-policy and technical-procedural. The first refers to the need to create a regulatory framework that includes unified policy and priorities ensuring effective communication across all stakeholders and to build local (state-academic integration) and international partnerships to make HRTUDP functionally applied. Furthermore, political influence on the decision-making and planning process should be prevented. For the technical-procedural track, it is indispensable to:

1) Ingrain the concepts, practices, and tools of health research, evidence-based decision-making, and knowledge-informed policy among decision-makers across sectors and also the concepts and approaches of knowledge transfer and dissemination among researchers, as previous studies have clearly emphasized [15–17];
2) Create dynamic communication channels between research and policy communities through knowledge-policy forums or centres, journals clubs, policy workshops, and national policy briefs and summaries; and
3) Encourage dissemination and distribution of research and knowledge through creative technological-institutional and national well-linked channels.

Lastly, the most important enabling opportunities to be exploited are existing bodies, initiatives, and previous attempts to push towards developed and capable HRTUDP, among them: the Palestinian National Institute of Public Health, The Supreme Palestinian Health Council, Scientific Research Council, Palestinian Council for Health Research, the Lancet annual conference, Lancet Palestinian Health Alliance, and other relevant stakeholders.

## Discussion

This study dealt with one of the most important pillars of the HRS [30]: exploring the system’s capacities in Palestine. As the HRS exists in a complex and diverse context [31, 32] and is of increasing concern to health policy-makers, providers, and others [7, 9, 33], the findings of this analysis are expected to contribute to the understanding of capacity components of the HRS in Palestine. This, in turn, will increase the potential for a successful and effective HRS based on active participation and well-strengthened capacity.

The findings of this study indicate that the level of research quality and compliance with good health research standards is still insufficient and needs more attention. A previous study found that, in Palestine, the subject of research capacity is below satisfactory [26], although research quantity in the wider Middle East and North Africa region is increasing and the quality of health research is improving [34]. The reasons for the low level of research quality and non-compliance with health research standards in Palestine can be related to institutional and environmental challenges. These include the lack of cohesive policies and priorities, lack of capacity and resources, and lack of institutional quality and ethical reviews, as one study proved [16]. Other gaps are caused by the lack of researchers’ knowledge and international exposure to health research quality standards and expertise. This, however, has been refuted by a study which indicated that international collaboration in research output was reported [24]. Further issues include the shortage of trusted and high-quality local journals, individualism instead of interdisciplinarity in research production, and, more importantly, the curriculum of health and medical schools not inherently addressing the fundamentals of scientific research which is required to be tackled to develop the students’ and graduates’ skills and knowledge.

The analysis of participants’ responses across both methods, interviews and FGDs, revealed four practical steps that may improve HRQS. These steps need to be applied concurrently and in a complementary approach. This means the creation of a policy that encourages all stakeholders to advance quality and standardization of research is essential and may lead to other strengthening actions such as communication mechanisms to improve exposure to international research societies, adapting and adopting standards, and improving the ethics review based on these standards as a key step of research process. The following are the steps that contribute to better quality of research:

1) A national policy that prioritizes the quality of health research.
2) Technological mechanisms such as interactive electronic platforms and hubs that can link external health research knowledge and expertise with the local research community [24].
3) The creation of guidelines for improving HRQS, embedding these guidelines into health schools’ curricula, and integrating them into capacity building programmes to develop researchers’ skills and capabilities. One example is the CONsolidated Standards of Reporting Trials (CONSORT) [26].
4) Strengthening health research ethical and technical reviews, evaluations, and follow-ups [16]. Embedding such foci in the education and training of researchers is a central activity for increasing the quality value of health research in Palestine.

The current study further evidently revealed several constraints to HRKTD. These include, for example, the fact that knowledge generally, and research outputs in particular, are not disseminated regularly and appropriately. Consequently, the evidence diffusion process remains weak mainly due to inadequate utilization and demand for research [35]. A comparable study showed that building HRSs to support HRKTD for improved health is one of the major challenges across the region [11].

Several impediments to good practice of HRKTD were identified, including:

1) Shortage in the culture of HRKTD among seniors at the high-management levels and research managers and researchers on HRKTD or evidence-informed policy-making concepts, and even research culture [17, 36].
2) HRKTD mechanisms and tools are lacking such as platforms, forums, peer-review journals, press releases, policy briefs, and libraries such as those recommended by Hinari Access to Research for Health Programme [37] and WHO [38]. Furthermore, HRKTD practices are often limited to the micro-institutional and individual levels, rather than through a systematic and inclusive national approach [35].
3) Health research is carried out for personal purposes or for donors’ agendas, with the result that the publication and dissemination of findings are often either missing or neglected.
4) In line with previous literature, this study emphasized the absence of a regulatory framework such as a body and policy, inadequate resources, and local and external poor coordination. This creates confusion in research production, dissemination, and utilization. Finally:
5) Difficulties related to data quality and availability, whether vital national statistics or data collected from research, as well as the conditions of borders blockage and movement obstacles that prevent the flow of researchers and materials to and from Palestine. Another study identified further gaps, such as the low level of engagement in the HRKTD activities due to the little support available in the HRKTD environment, including the lack of incentives [35].

These impediments were specifically expressed due to the unique case of Palestine which may or may not exist in other settings. Three trajectories featured these obstacles: individual, institutional, and environmental factors. Firstly, individually, research produced stops at the step of sharing. Usually these research efforts stem from sometimes unnecessary priorities and are unembraced institutionally. Secondly, institutional factors related to the unreinforced culture on research that prevails in institutions and about the HRKTD in particular between departments, institutions, and across sectors. Thirdly, environmental and national factors related to the absence of frameworks that regulate all health research activities, which leads to the absence of appropriate and systematic dissemination mechanisms, including restrictions imposed on many Palestinian institutions in terms of logistical, technical, communication, and financial resources. These restrict the ability to perform all stages of the research process effectively.

For better HRKTD, as highlighted previously in the HRS stakeholder analysis, there is a need to increase political level involvement and advance a regulatory framework for health research. Three main Palestinian entities, the Ministry of Health, Ministry of Higher Education and Scientific Research, and the Palestinian National Institute of Public Health, can be a national body responsible for advancing this framework. This consortium requires adequate representation from the NGOs, academic institutions, relevant independent national bodies and councils, and international partners. This move should make it possible to define the stakeholders’ roles to improve coordination and the concepts of research and HRKTD among decision-makers and researchers alike [4, 11, 35, 39]. More investment is necessary to establish HRKTD strategy including use of innovative technology, for example, a national archive for science data [37] or national platforms such as knowledge-informed policy [40], knowledge for policy, strategic policy platforms, or research decision-making centres or labs. This strategy should embrace the WHO model for HRKTD [38]. The strengthening of Palestinian national and institutional databases is needed to improve quality, organization, accessibility, and transparency, and enhance the local and international partnerships and collaboration in health research production and dissemination [7].

The study found HRTUDP, a central concept emphasized by the WHO Eastern Mediterranean Regional Office in its strategic directions research for health, to be a pivotal tool for health development and informing health policy improvement [35]. HRTUDP was first demonstrated by the Canadian Institute of Health Research to bridge the knowledge-practice gap and is now widely used with interchangeable terms in the literature (e.g. knowledge transfer, research utilization, evidence implementation) [41]. HRTUDP in Palestine exists under constrained circumstances and has, so far, not been a key tool in the decision-making process in the country’s health system. This study identifies knowledge and policy gaps and it recommends enhancing the research-policy interface [35]. The problem of an unsupportive culture for research to become acknowledged should be addressed, where decisions or policies are mostly not evidence- and knowledge-based, and decision-makers are not knowledge-oriented [42]. This is also consistent with AlKhaldi et al., on the deficient conceptualization level on health research or HRS at large [17]. In return, a study carried out in the Middle East and North Africa region denies the presence of negative attitudes among policymakers towards research evidence, its use, and benefits in practice. It calls for fostering evidence-informed policy-making by establishing a clear understanding of the national context in which policy decisions are made [35].

Other identified technical constraints are a deficit in trust and inconsistent relations [43] between the knowledge-producers and the decision-makers, which weakens knowledge diffusion. Moreover, academic knowledge production was found to be an area of low investment by the state. A further limitation related to health research quality and credibility is research deficiency in addressing real priorities with plenty of descriptive studies compared to a paucity of experimental studies, which offer more trustworthy evidence. This is clearly emphasized by a similar study [44]. Finally, with the absence of good health research governance, policies, and priorities, scarcity of resources, and institutional management changes, and political instability as literature demonstrated [8, 23, 35, 45–47], knowledge and research-informed policy-making will remain impossible unless these fundamental hindering issues are addressed. Therefore, substantive structural and technical-procedural changes should be implemented to promote knowledge translation and decision-making practices and to eliminate preference-based decisions, whereby the health systems could eventually be strengthened [43, 48–50].

These are the changes and improvements suggested:

First: Urgent synergized efforts to establish a well-structured HRS, involving and organizing all health research components, including HRTUDP. This is largely consistent with the debate at the Global Forum 2015 which attributed the low uptake of evidence partly to weak governance and sub-optimal collaboration and engagement among research, industry, policy-making, and community societies [43].

Second: The concept of HRTUDP and evidence and knowledge-based practices need to be entrenched among the health system’s decision-makers and reseachers, including health research, HRS values, goals, and stewardship functions, HRQS, and HRKTD concepts.

Third: Building knowledge translation strategies consisting of effective communication channels and interactive integration spaces mandated by the Ministry of Health and academia, as proposed in HRKTD, such as national health research or knowledge-policy networks, forums [40], models [51], journals, labs, Hackathons, centres, clubs, policy briefs magazines, and media releases [38, 43]. Fourth: Maintaining synergy and dynamic mechanisms between HRTUDP and HRKTD as they both complement each other [44].

Fifth: Capacity building and education programmes on HRKTD should be provided in collaboration with local and international partners. In Palestine, some active bodies, such as local universities, the Palestinian National Institute of Public Health, and different initiatives, such as The Lancet Palestinian Health Alliance^2^, are likely to be driving forces to achieve that.

The study limitations can be summarized as follows:

1) The substantial knowledge gap of relevant literature on the subject as well as the lack of data availability, quality, organization, and accessibility;
2) Time constraint for an even more comprehensive analysis, linked to the restrictions on the freedom of movement of the research team as a result of the closure and security checkpoints; and
3) The constant environmental and political fluctuations and institutional changes.

Consequently, the present study proposes further research in the following key areas:

1) A national need assessment study or quantitative study may be useful to precisely determine and assess the HRSC currently available such as practices, assets, resources, and facilities at the individual, institutional, and national levels.
2) There is a need to examine, perhaps by using observation or case study methods, specific knowledge transfer and application practices.

### Conclusion

There is a growing global and regional concern about advancing the core capacities of HRS, HRQS, HRKTD, and HRTUDP. These capacities relate to the ultimate goal of the HRS, which is the generation of scientific knowledge and the promotion of this knowledge to improve health system performance, achieve Sustainable Development Goals, and ensure the healthy lives and well-being of the population. The process of health research and knowledge production starts with designing and implementing rigorous decision-relevant research, publishing and sharing its outputs, and translating these outputs into health policy interventions and practices. To achieve this, there is an emphasis on the need to build well-functioning HRS. This system needs to be strengthened through deep analysis and understanding of the system's pillars, mainly health research quality and knowledge transfer and translation.

The study has significance stemming from the importance of the subject under investigation, which has only been minimally assessed in Palestine and in the Middle East and North Africa region. The study offers an in-depth description of the current pattern of the three key capacities, the identification of the major gaps hindering the advancement of HRS capacities, and suggestions to strengthen the three capacities of HRS in Palestine. This study could be an essential scientific reference, a fresh research attempt to draw the attention of local and regional health decision-makers, health professionals, and researchers. In summary, the study revealed that the overall pattern of Palestinian HRS capacities is characterized by considerable systematic weakness. The issue of HRSCS deserves to be of high priority for the state and non-state stakeholders to pay attention and invest to enable these capacities at the individual, institutional, and national levels.

The study concludes that weaknesses in policies, resources, institutional capacity, and individual orientation, as well as in the education curricula are at the bottom line of the low level of HRQS. A national policy, which embeds and institutionalizes international guidelines in the settings of teaching, research, and practice, has the capacity to develop education programmes and improves professionals’ and researchers’ understanding of research quality topics. Furthermore, it also promotes capacity and resources at the individual, institutional, and national levels.

Health research and knowledge outputs are not disseminated properly and systematically due to the lack of regulatory framework. Such a framework needs to be established as a stewardship structure in Palestine or any country by the concerned ministries of health, education, justice and their partners. The mandate of this structure is to oversee all functions of research governance, ethics, funding, and technicalities. Other reasons for weak dessimiantion are the low understanding level and culture among seniors and researchers on knowledge sharing and transfer, shortage of HRKTD mechanisms and coordination means, lack of data quality and availability, and environmental restrictions. Political participation, knowledge transfer and dessimination strategy, and communication mechanisms such as establishing a national observatory or platform for both HRKTD and HRTUDP, and local and international partnerships are substantial actions for successful, dynamic HRKTD. Knowlege translation and utlization into decision and policy, as a central concept for health development and policy improvement, is remarkably constrained in Palestine and it is not an essential decision-making methodology. Knowledge producers’ and users’ disconnection, culture, health research credibility, applied research, and governance and resources and management and political changes are behind ineffective HRTUDP, where an organizing framework, entrenching its concepts and strategies and mechanisms and structurally linked with HRKTD needs to be implemented. Ultimately, although our study is specific to the Palestinian cause, implications for the wider context are apparent and particularly relevant for developing countries and the region. There is a need to reemphasize the perspective of thinking outside the box by focusing on strengthening HRS capacity to ensure good practice for high-quality knowledge and evidence uptake in Palestine and in the region. Actions identified in this study, whether at the individual, institutional, country, or regional level or beyond, should be addressed through political commitment, national consensus, a unified governing structure and system and HRSCS strategy as well as partnerships and collaboration.

## Data Availability

The original contributions presented in the study are included in the article/supplementary material, further inquiries can be directed to the corresponding author.

## Footnotes

1 K2P Centre in the Middle East founded by the Faculty of Health Sciences (FHS) at the American University of Beirut, to bridge the gap between science, policy, and politics by making research evidence more accessible to a broader range of stakeholders, building institutional capacities for evidence-informed policymaking, and seizing opportunities to advocate and influence policy outcomes. https://www.aub.edu.lb/k2p/pages/default.aspx.

2 The Lancet Palestinian Health Alliance (LPHA) was established in 2009 to be an informal network of Palestinian, regional, and international colleagues researching the health of Palestinians. Ten annual scientific conferences have been held, with 748 research presentations involving more than 1,000 co-authors. The LPHA has no representative or political purpose and prefers to speak only via the evidence presented in research studies on Palestine. https://www.thelancet.com/journals/lancet/article/PIIS0140-6736(21)00185-9/fulltext.

## Abbreviations

CONSORT, consolidated standards of reporting trials; FGDs, focus group discussions; HRKTD, health research knowledge transfer and dissemination; HRQS, health research quality and standardization; HRS, health research system; HRSC/HRSCS, health research system capacity/HRS capacity strengthening; HRTUDP, health research translation and utilization into decisions and policies; IDIs, in-depth interviews; NGOs, Non-governmental Organizations; WHO/EMRO, World Health Organization/Eastern Mediterranean Regional Office.
